# Pathogenesis and Clinical Significance of In-Stent Restenosis in Patients with Diabetes

**DOI:** 10.3390/ijerph182211970

**Published:** 2021-11-15

**Authors:** Grzegorz K. Jakubiak, Natalia Pawlas, Grzegorz Cieślar, Agata Stanek

**Affiliations:** 1 Department and Clinic of Internal Medicine, Angiology, and Physical Medicine, Faculty of Medical Sciences in Zabrze, Medical University of Silesia, Batorego 15 St., 41-902 Bytom, Poland; grzegorz.k.jakubiak@gmail.com (G.K.J.); cieslar1@tlen.pl (G.C.); 2 Department of Pharmacology, Faculty of Medical Sciences in Zabrze, Medical University of Silesia, Jordana 38 St., 41-800 Zabrze, Poland; natalia.pawlas@sum.edu.pl

**Keywords:** diabetes mellitus, atherosclerosis, percutaneous transluminal angioplasty, peripheral arterial disease, restenosis, in-stent restenosis

## Abstract

Diabetes mellitus (DM) is a strong risk factor for the development of cardiovascular diseases such as coronary heart disease, cerebrovascular disease, and peripheral arterial disease (PAD). In the population of people living with DM, PAD is characterised by multi-level atherosclerotic lesions as well as greater involvement of the arteries below the knee. DM is also a factor that significantly increases the risk of lower limb amputation. Percutaneous balloon angioplasty with or without stent implantation is an important method of the treatment for atherosclerotic cardiovascular diseases, but restenosis is a factor limiting its long-term effectiveness. The pathogenesis of atherosclerosis in the course of DM differs slightly from that in the general population. In the population of people living with DM, more attention is drawn to such factors as inflammation, endothelial dysfunction, platelet dysfunction, blood rheological properties, hypercoagulability, and additional factors stimulating vascular smooth muscle cell proliferation. DM is a risk factor for restenosis. The purpose of this paper is to provide a review of the literature and to present the most important information on the current state of knowledge on mechanisms and the clinical significance of restenosis and in-stent restenosis in patients with DM, especially in association with the endovascular treatment of PAD. The role of such processes as inflammation, neointimal hyperplasia and neoatherosclerosis, allergy, resistance to antimitotic drugs used for coating stents and balloons, genetic factors, and technical and mechanical factors are discussed. The information on restenosis collected in this publication may be helpful in planning further research in this field, which may contribute to the formulation of more and more precise recommendations for the clinical practice.

## 1. Introduction

### 1.1. Diabetes Mellitus and Atherosclerotic Cardiovascular Disease

Diabetes mellitus (DM) and its complications constitute one of the most important problems in public health worldwide. 382 million people were estimated to have DM in 2013 and this number is expected to increase to 592 million by 2035 [1]. According to another publication, approximately 462 million individuals were affected by type 2 DM in 2017 [2]. Type 1 DM (T1DM) is responsible for approximately 5–10% of cases, and autoimmune damage to beta cells is responsible for its development, leading to insulin deficiency. Type 2 DM (T2DM) accounts for approximately 90–95% of cases, and insulin resistance, as well as beta cell dysfunction, play an important role in its pathogenesis. Other types of DM include gestational DM and secondary DM [3]. DM is a significant risk factor for the development of atherosclerotic cardiovascular diseases such as coronary heart disease (CHD), cerebrovascular disease, and peripheral arterial disease (PAD) [4]. It should be noted that cardiovascular diseases represent the leading cause of morbidity and mortality in patients with DM [5], and on the other hand, DM worsens the prognosis in patients with CVD [4], so improving the knowledge about pathogenesis and the development of more effective methods for diagnosis and treatment of cardiovascular diseases play a crucial role.

### 1.2. Peripheral Arterial Disease in the Course of Diabetes

DM itself accelerates the progression of atherosclerosis. Therefore, patients with DM are characterised by the higher incidence of PAD and the appearance of the comorbidities mentioned above. Furthermore, the severity of PAD increases in patients with coexisting DM and it is associated with a higher rate of amputation in this group [6]. The risk of major amputation in patients with DM is five to ten times higher than in those without DM [7]. The prevalence of PAD in patients with DM over 40 years of age was estimated at 20% [8]. In patients with pre-existing DM, the increased risk of PAD is associated with duration of DM, peripheral neuropathy, and age [9,10]. Furthermore, in patients with DM, for every 1% increase in the value of percentage of glycated haemoglobin, there is a corresponding 26% increase in the risk of the developing PAD [11]. DM is also associated with more severe below-the-knee PAD [12].

PAD is a significant medical problem that affects approximately 5.3% of the inhabitants of the European continent (around 40 million individuals). A typical symptom is intermittent claudication, which limits the functional capacity of patients and contributes to the deterioration of their quality of life. It also generates an economic burden for healthcare systems [7]. The most severe form of PAD is critical limb ischemia (CLI), which is characterised by pain at rest or the presence of ulceration and tissue necrosis. It can lead to amputation [13]. The association between PAD and frailty syndrome in patients living with DM has recently been widely described [14].

### 1.3. Percutaneous Transluminal Angioplasty with or without Stent Implantation

Percutaneous transluminal angioplasty (PTA) with or without stent implantation plays an important role in the treatment of PAD [15], as well as CHD, taking into account stable and acute coronary syndromes (within the coronary arteries, the procedure for angioplasty is called percutaneous coronary intervention, PCI) [16] and extracranial carotid atherosclerotic disease (but endarterectomy remains thought to be the gold standard) [17]. Restenosis is a major concern that limits the long-term effectiveness of the percutaneous revascularisation. It consists of the relapse of stenosis at the site where angioplasty has been performed, and it may cause a necessity for reintervention [18]. According to the actual guidelines of the European Society for Vascular Medicine in the endovascular treatment of lower limb ischemia, PTA with optional stent implantation is recommended [15]. The decision on stent implantation in clinical practice should be made taking into account mainly the effect of PTA, residual stenosis, and the presence of a significant dissection of the arterial wall.

Stents may be classified based on such criteria as mechanism of expansion (self-expanding or balloon-expandable), composition (stainless steel, tantalum, nitinol, cobalt-based alloy, inert coating, active coating, or biodegradable), and design (slotted tube, mesh structure, ring, coil, custom design, or multi-design) [19]. From the point of view of clinical practice, it is worth distinguishing bare-metal stents (BMSs) from drug-eluting stents (DESs). DESs are coated by one of the antimitotic agents, such as sirolimus, everolimus, zotarolimus, or paclitaxel [20]. Although the introduction of DESs was an important moment in improving the long-term efficacy of percutaneous revascularisation, restenosis remains a significant problem [21]. Diabetes mellitus is a strong independent risk factor for restenosis both after non-stent balloon angioplasty and PTA with stent implantation, both in terms of the use of BMS and DES [22,23].

### 1.4. Purpose of This Paper

The purpose of this paper is to provide a review of the literature and to present the most important information on the current state of knowledge of mechanisms and the clinical significance of restenosis and in-stent restenosis (ISR) in patients with diabetes, especially in association with the endovascular treatment of PAD.

## 2. General Mechanism of Restenosis

Balloon dilatation and stent implantation are associated with vascular injury followed by repair processes that include endothelialisation and neointimal formation [24]. The vascular wall injury causes activation of the inflammatory response to mechanical stretch, endothelial denudation, and subintimal haemorrhage [25]. It is worth mentioning that low-grade inflammation is a common feature in patients with T2DM [26].

Local and systemic inflammation plays a key role in the activation of the proliferative process, which consists of the proliferation, migration, and differentiation of vascular smooth muscle cells (VSMCs) as well as the migration of matrix metalloproteinases (MMPs), DNA replication, and extracellular matrix synthesis [25]. Endothelial progenitor cells were shown to take part in the stimulation of the proliferation and migration of VSMCs, which leads to an increase in their neointimal accumulation following vascular wall injury [27]. All these processes result in neointimal hyperplasia [28]. Further growth of neointima leads to a relapse of the narrowing of the vessel lumen (restenosis), and this process is called neoatherosclerosis [29]. The most fundamental process that characterises neoatherosclerosis is the accumulation of lipid-laden macrophages that form foam cells within neointima. It can be further followed by necrotic core formation, calcification, or thrombosis. Romero et al. published a valuable review that describes the pathology of the neoatherosclerosis process [30].

The most important differences and similarities between neoatherosclerosis and atherosclerosis in native arteries are summarised in Table 1 and between neoatherosclerosis related to ISR in BMSs and DESs in Table 2.

### Characteristic Features of the Development of Atherosclerosis in the Course of Diabetes

Thiruvoipati et al. presented a detailed description of the mechanisms that lead to the development of atherosclerosis, especially in the context of PAD, in patients with DM, which is partially different from the development of atherosclerosis in the population without DM. Similar mechanisms may also to some extent be related to the phenomenon of ISR in this population [12]. A summary of these mechanisms is shown in Table 3.

## 3. Inflammation

### 3.1. C-Reactive Protein and Proinflammatory Cytokines

Inflammation plays a crucial role in the process of atherogenesis and restenosis. Moreover, DM has been shown to be associated with elevated levels of CRP and proinflammatory cytokines, suggesting a role for the inflammatory process in diabetogenesis [44,45].

The role of inflammation in restenosis is partially confirmed by systemic inflammatory markers, but the scientific data is not unambiguous. Previous studies suggested that, although the elevated level of CRP predicts ISR in BMS [46,47], there is no association between abnormal CRP concentration and ISR in DES [48,49]. The literature data on the concentration of CRP and other inflammatory markers in patients treated with BMS or DES have shown some discrepancies. It was documented that, 24 h after PCI, the increase in inflammatory markers (CRP and interleukins such as IL-1Ra and IL-6) is similar in the DES-treated group and in the BMS-treated group [49]. It was shown that, 48 h and 72 h after PCI, DES-treated individuals have significantly lower CRP concentrations than BMS-treated individuals (5.9 ± 4.9 vs. 13.4 ± 14.7 mg/L after 48 h respectively, *p* < 0.01, and 5.4 ± 3.9 vs. 16.7 ± 19.8 mg/L after 72 h respectively, *p* < 0.01) [50]. Taking into account more actual data and the use of high-sensitivity CRP (hs-CRP) instead of CRP, the relationship between systemic inflammatory parameters and the incidence of ISR seems to be more complicated.

According to a meta-analysis of seven studies, preprocedural abnormal CRP level did not correlate with the risk of ISR in patients with ST-elevation myocardial infarction (STEMI) treated by PCI, although it is associated with the significantly higher in-hospital and follow-up all-cause mortality [51]. No significant correlation between the concentration of such inflammatory markers as tumour necrosis factor alpha (TNF-α), transforming growth factor beta (TGF-β), IL-6, IL-8, IL-12, and CRP, and the risk of ISR in half-year follow-up was found in patients undergoing femoral PTA with stent implantation due to PAD [52].

The elevated postoperative hs-CRP level is associated with an increased risk of ISR in one-year follow-up in patients who have undergone PCI with DES implantation [53]. In another study, the average hs-CRP level was shown to be significantly higher in patients in whom ISR had occurred. The authors of the publication also calculated that 2.64 mg/dL was the cut-off value below which diabetes was the only significant factor found to predispose a patient to ISR, while when it was at least at that level, diabetes, triglyceride blood concentration, and type of stent were factors associated with the development of ISR [54]. In a meta-analysis of six prospective observational trials, it was confirmed that the higher level of hs-CRP is associated with a significantly increased risk of ISR (OR 1.16, 95% CI 1.01–1.30; *p* < 0.05) [55].

CRP and inflammatory cytokines such as TNF-α and IL-6 are directly involved in the process of atherogenesis, according to the mechanisms shown in Table 4.

The increased C-reactive protein-to-albumin ratio (CAR) was shown to be a valuable inflammatory marker predicting ISR in patients undergoing iliac artery stenting (HR 2.66, 95% CI 1.66–4.25; *p* < 0.01) [56], as well as in patients with STEMI (OR 2.289, 95% CI 1.056–4.959; *p* = 0.036) [57].

### 3.2. Complement System

The activation of the complement system may also take part in the pathogenesis of restenosis. It was shown that a higher C5a level is associated with an increased risk of restenosis in patients with PAD who had undergone PTA of the superficial femoral artery (SFA). C5a concentration was measured at baseline and eight hours after the procedure. It should be noted that, in this study, restenosis was diagnosed not with angiography but with duplex ultrasound [58]. Previously, elevated C5a concentration was shown to be associated with increased cardiovascular risk in patients with advanced atherosclerosis [59].

The upregulation of the complement system plays an important role in the development of T1DM and T2DM, as well as in the pathogenesis of the complications of DM [60]. It was documented that the complement system plays a significant role in the development of diabetic kidney disease [61,62,63], retinopathy [64], and periodontal destruction in patients with DM [65]. Interestingly, the mechanism by which the complement system promotes the development of diabetic kidney disease is the activating of the signal transducer and activator of transcription 3 (STAT3) pathway [63]. The crucial role of STAT3 activation in vascular disease (also taking into account restenosis) has been elucidated [66].

The complement system is also involved in the development of macrovascular complications of DM due to its involvement in the atherosclerosis process [60]. The terminal complement C5b-9 complex was shown to play a crucial role in the activation and proliferation of VSMCs and endothelial cells (ECs) [67]. The blood concentration of C3 was shown to be significantly higher in PAD patients than in healthy individuals. C3 and C4 levels were inversely correlated with the ankle–brachial index (ABI) [68]. The role of the complement system in association with the genotypes of mannose-binding lectin (MBL) 2 in restenosis was suggested, especially within carotid arteries [69]. MBL is the main protein in the lectin complement pathway. The role of MBL in ischemia and reperfusion injury in different vascular beds has been also elucidated [70]. In a study carried out on apolipoprotein E-deficient mice, it was shown that the deficiency of the second receptor for complement anaphylatoxin C5a (C5aR2) is associated with attenuated atherogenesis and neointimal formation. Plaques in C5aR2-deficient mice showed significantly reduced expression of the first C5a receptor (C5aR1) and TNF-α, as well as the vascular cell adhesion molecule-1 (VCAM-1). Moreover, blocking C5aR1 in *C5ar2*^−/−^/*Apoe*^−/−^ mice led to a further reduction in inflammation and neointimal formation [71]. The expression of C5aR2 was shown to correlate with the expression of proinflammatory cytokines in advanced human atherosclerotic plaques [72].

According to our best knowledge, there are no papers directly connecting the activation of the complement system with restenosis in patients with DM undergoing PTA and stenting due to PAD. It would be an interesting and promising direction for future research.

### 3.3. Inflammatory Cells

Hyperglycaemia has been shown to promote leukocytosis (especially in terms of monocytes and neutrophils) [73]. Some parameters associated with inflammatory cells in peripheral blood were shown to have prognostic value in patients after the endovascular treatment of disease caused by atherosclerotic lesions.

In patients undergoing stent implantation to SFA, there is a positive correlation between the post-procedure medium platelet volume and the risk of ISR (*p* < 0.001). A pre- and post-procedure mean platelet volume difference no less than 1.5 fL was documented to be associated with an increased risk of ISR (OR 9.17, 95% CI 3.76–22.35; *p* < 0.001), similarly to the mean platelet volume difference ratio being no less than 17.9% (OR 7.68, 95% CI 3.19–18.49; *p* < 0.001) [74]. Furthermore, the neutrophil-to-lymphocyte ratio (NLR) after procedure of stent implantation to SFA, as well as the NLR change ratio, but not NLR before the procedure, may be useful in assessing of the risk of ISR. NLR after a procedure higher than 4.3 was shown to have 75% sensitivity and 76% specificity for the prediction of the occurrence of ISR [75]. According to Chang et al., the NLR cut-off point of 3.62 has a sensitivity and specificity of 73.4% and 80.4%, respectively, to predict the occurrence of ISR in the first year after the procedure in patients undergoing femoropopliteal stenting due to chronic total occlusion (CTO) [76]. In another study, the platelet-to-lymphocyte ratio (PLR) as well as NLR, but not the lymphocyte-to-monocyte ratio (LMR), was shown to predict the risk of ISR after infrainguinal PTA with stent implantation [77].

Interestingly, NLR after femoropopliteal PTA without stent implantation was shown to be significantly lower in patients treated with a drug-coated balloon (DCB) compared to patients treated with PTA with a uncoated balloon. Moreover, it was associated with a significantly higher primary patency rate after six months [78].

NLR was documented to increase among patients with DM and, in such patients, is independently associated with the prevalence and severity of CHD [79]. NLR was shown to be useful in assessing the risk of ISR after PCI with DES implantation in patients with T2DM [80].

## 4. Neointimal Hyperplasia and Neoatherosclerosis

### 4.1. Gap Junctions and Connexin 43

Gap junctions (GJs) are intercellular structures that allow for communication and the flow of small molecule substances such as ions, glutathione, glucose, cyclic adenosine monophosphate (cAMP), and inositol 1,4,5-trisphosphate (IP_3_). A single GJ is made up of connexons. Each connexon consists of six connexin molecules [81]. One of them is connexin 43 (Cx43). Cx43 occurs abundantly in GJs, and its role in the pathogenesis of neoplastic and cardiovascular diseases has been pointed out, although it is yet not fully understood [82,83]. Cx43 phosphorylated by mitogen-activated protein kinase (MAPK), through interaction with cyclin E, was documented to contribute significantly to the activation of platelet-derived growth factor (PDGF)-mediated VSMCs proliferation, which may be of key importance in the development of atherosclerosis as well as ISR [84]. Interestingly, simvastatin contributes to the reduction of Cx43 expression by inhibiting the effects of TNF-α and IL-18 on the upregulation of Cx43 within VSMCs. This is one of mechanisms that may explain the beneficial effect of statins in inhibiting atherogenesis [85]. Cao et al. conducted an interesting study on rabbits, the results of which suggest the involvement of Cx43 in the development of restenosis after iliac artery angioplasty. The authors concluded that PTA results in a local activation of the renin-angiotensin-aldosterone system, leading to the upregulation of Cx43 by the AT_1_ receptors for angiotensin II. As a result, intercellular information exchange is activated, which is associated with increased proliferation and migration of VSMCs and neointimal hyperplasia [86]. An association between Cx43 and resistance to paclitaxel, as well as some information about the influence of high blood glucose level on Cx43 regulation were described below.

### 4.2. Biochemical Pathways of Signal Transduction

Studies on VSMCs isolated from the aorta of wild-type (WT) and *Tnfrsf12a* knockout mice showed that tumour necrosis factor-like weak inducer of apoptosis (TWEAK) via the Fn14 receptor stimulates the proliferation of VSMCs. TWEAK/Fn14 is considered a potential target for treating ISR [87]. In vitro studies have shown that TWEAK/Fn14, through the canonical and non-canonical activation of the NF-κB factor, participates in the calcification of VSCMs, which is particularly important, inter alia, in the population of patients with DM [88]. More research is required on the possible clinical application of these findings in the treatment of ISR.

Studies in rats with streptozotocin-induced T1DM elucidated that insulin may contribute to neointimal hyperplasia through the phosphatidylinositol 3-kinase/Akt pathway (the PI3K/Akt pathway) after balloon angioplasty in carotid arteries [89]. The AMP-activated protein kinase/Nox4 signal pathway (the AMPK/Nox4 pathway) is also involved in the neointimal hyperplasia process, and combined therapy with linagliptin and metformin leads to a decrease in the formation of neointima in rats [90]. The translocator protein of the outer mitochondrial membrane takes a part in neointimal hyperplasia via the activation of protein kinase G (PKG) in rats with T2DM [91].

Expression of SIK3, a member of the salt-inducible kinases (SIKs), which is a subfamily of the AMPK family, was shown to be lower in VSMCs with a contractile phenotype and higher in VSMCs with a proliferating phenotype. SIK3 is overexpressed in neointimal lesions in vitro. SIK3 inactivation probably supresses the proliferation of VSMCs by down-regulating PKA-cAMP response element binding protein (PKA-CREB), PKB-CREB, and CRT3 (CREB-regulated transcriptional coactivator 3) signalling [92].

It was suggested that microRNA molecules, such as miR-181b and miR-204, may have a therapeutic potential in preventing the development of atherosclerotic plaque and restenosis through the inhibition of the proliferation and migration of VSMCs mediated by the β-arrestin-2/extracellular signal-regulated protein kinase pathway (the β-ARR2/p-ERK1/2 pathway) [93].

### 4.3. Bone Morphogenetic Protein

As it is known, DM is a condition that predisposes patients to increased vascular stiffness as a result of the calcification of the middle layer of the artery wall [15], which in turn predisposes patients to the development of restenosis, and one of the factors responsible for this is the overexpression of bone morphogenetic protein-2 (BMP-2) in VSCMs [94].

Recent studies in mouse models have shown that platelet bone morphogenetic protein-4 (BMP-4) may play an important role in the neointimal formation process after vascular injury. The expression of P-selectin was shown to be reduced in platelets from *Bmp4^Plt−/−^* mice, and fewer platelet-leukocyte aggregate formations were found than in control mice [95].

### 4.4. Alternative Splicing and Epigenetic Mechanisms in VSMCs

It was suggested that epigenetic mechanisms may play an important role in the pathogenesis of vascular complications in the course of DM. DNA methylation, histone modification, and non-coding RNAs are epigenetic mechanisms that play a role the pathogenesis of ISR [96]. Histone demethylase KDM3a promotes the proliferation and migration of VSMCs involved in vascular neointimal hyperplasia in diabetic rats. It was suggested that epigenetic mechanisms may be a target for therapy and the prevention of restenosis in future [97]. In a rodent model of insulin resistance and DM, oxidised LDL was shown to suppress the expression of gene encoding Src homology-2-containing protein tyrosine phosphatase (SHP-1) by the increased DNA methylation of the *Shp-1* promoter, which leads to excessive VSMCs proliferation and intimal hyperplasia [98].

Green et al. noted the role of alternative splicing in the phenotypic plasticity of VSMCs and that, when this plasticity became pathological, VSMCs played an important role in the development of atherosclerosis and restenosis [99].

### 4.5. Ischemia-Reperfusion Injury in the Arterial Wall Associated with PTA

The ischemia-reperfusion injury to the artery wall that occurs during PTA plays a role in the formation of neointima. Hypoxia-inducible factors (HIFs) are transcription factors that play an important role in adaptation to hypoxic conditions. In studies on *Apoe^−/−^* mice, the local inhibition of HIF activity was found to reduce the formation of injury-induced neointima [100]. The activity of phosphoglycerate kinase 1 (PGK1), a glycolytic enzyme, is associated with cell survival under hypoxia. As a result of PTA-induced arterial wall injury, PGK1 expression has been shown to increase, and the inhibition of PGK1 activity inhibits VSMCs migration and proliferation, leading to the suppression of neointima formation [101].

### 4.6. Neointima Formation and Neoatherosclerosis in Patients with and without Diabetes

Gao et al. found that the incidence of neoatherosclerosis after PCI with DES implantation is comparable in patients with and without DM. The evaluation was performed using optical coherent tomography. There was a higher incidence of neovascularisation in neoatherosclerotic lesions in the diabetic population. Suboptimal blood glucose level was associated with an icreased incidence of thin-cap fibroatheroma [102].

An optical coherence tomography study showed that, in patients with DM, the mean thickness of the neointima is significantly higher than in patients without DM (160 (90–240) µm vs. 110 (60–220) µm, *p* = 0.048). The study concerned people after the implantation of second-generation DES due to CHD [103]. Interestingly, empagliflozin has been shown to reduce neointima thickness assessed by optical coherence tomography in post-PCI patients with second-generation DES implantation [104].

## 5. Allergy

Allergic inflammation plays a part in the pathogenesis of coronary plaque progression and coronary plaque instability, as well as adverse events associated with stent implantation [105]. The total number of eosinophils in peripheral blood counted six weeks after PCI with DES implantation was shown to be significantly higher in patients with ISR than without ISR (267 ± 132 cells/μL vs. 174 ± 133 cells/μL, *p* < 0.01) [106]. The blood concentration of eosinophil cationic protein (ECP) was shown to be significantly higher in patients with ISR than in the group without ISR after PCI with DES implantation (17.7 ng/mL vs. 9.0 ng/mL, *p* = 0.017), with no significant difference in the blood level of hs-CRP and immunoglobulin E (IgE) [107]. ECP level correlates with the occurrence of such events as cardiac death, recurrent myocardial infarction, or clinically driven target lesion revascularisation in patients undergoing PCI with the implantation of first-generation DES [108]. In another study, a basal ECP level of more than 11 g/L was the only significant predictor of major adverse cardiac events after BMS implantation (HR 3.5, 95% CI 1.1–10.4; *p* = 0.03) [109]. Eosinophils were shown to be involved in inflammatory infiltration in histopathological samples taken from patients with ISR in DESs and BMSs [110].

Niccoli et al. showed, using optical coherence tomography, that patients with a high level of ECP had a higher neointimal burden compared to patients with a low level of ECP [111]. The meta-analysis performed by Gong et al. confirmed that allergy to stent material worsens the prognosis of patients after coronary stent implantation [112]. Interstitial eosinophil infiltration has been proven to have prognostic value in patients with diabetic nephropathy [113]. In patients with T1DM, eosinophils have been shown to be characterised by increased activity assessed by features such as a high level of myeloid alpha-defensins and myeloperoxidase [114]. Interactions between eosinophils and platelets are involved in the pathogenesis of atherosclerosis [115]. The mean intima-media thickness (IMT) measured in the common carotid artery but not the maximum IMT correlates with the eosinophils count in peripheral blood in Japanese individuals with T2DM [116]. An interesting case report of an 83-year-old Japanese male with ISR exacerbated by drug-induced severe eosinophilia (maximum was 6500/μL, 48% of total white blood cell count) after second-generation DES implantation has been described [117].

The assessment of the role of allergic inflammation in the development of ISR, as well as in patients living with DM, could be an interesting topic for future research.

## 6. Drug Resistance

An interesting issue is the influence of individual factors determining resistance to drugs coating DESs on the occurrence of ISR. The clinical relevance of this possible mechanism contributing to ISR after DES implantation is currently poor understood [25].

### 6.1. Paclitaxel

A detailed review summarising the pharmacological properties of paclitaxel has been published recently by Gallego-Jara et al. Paclitaxel is an antimitotic drug whose main mechanism of action is binding tubulin and inhibiting the disassembly of microtubules. It also causes cell death through the stimulation of apoptosis. Paclitaxel has been shown to dysregulate the toll-like receptor 4 (TLR4) and the NOD-, LRR-, and pyrin domain-containing protein 3 (NLRP3) inflammasome [118]. In a noteworthy study, TLR4 was shown to be a target for neutrophil elastase by which this enzyme takes a part in neointimal formation [119].

In a study carried out on a mouse model of pancreatic adenocarcinoma, it was documented that T2DM induces microbiome dysbiosis causing poor response to gemcitabine/paclitaxel, which is considered to be the standard therapy for this cancer [120]. Therefore, such results give rise to a reasonable suspicion that T2DM may, in a similar mechanism, contribute to the reduction in the effectiveness of paclitaxel in preventing restenosis. This is an interesting direction for future research.

The role of Cx43 in neointimal hyperplasia was mentioned above. The overexpression of Cx43 was shown to suppress the expression of genes associated with resistance to paclitaxel, such as genes encoding breast cancer resistance protein (BCRP), transcriptional factor Txr-1, α-tubulin, and β-tubulin, and, on the other hand, it promotes the expression of the genes of apoptosis such as *Tsp-1* and *Bcl-2*. The overexpression of Cx43 can also increase the potency of paclitaxel by direct binding to β-tubulin [121]. Previously, it was documented that the phosphorylation of Cx43 by PKC leads to a decrease in the expression of Cx43 and that this effect makes cells of ovarian cancer more chemosensitive (paclitaxel and cisplatin have been taken into consideration) [122]. In a study on glomerular mesangial cells isolated from rats, it was shown that a high glucose level induces a decrease in the expression of Cx43. It is associated with hyperglycaemia-induced hypertrophy, and the modulation of the PTEN/Akt/mTOR pathway plays a role in this process. In this study, a “high glucose level” was defined as 30 mM [123]. From the point of view of human pathophysiology, this value of glycaemia is very large but is often observed in the clinical practice in patients with decompensated or untreated DM. On the other hand, rat fibroblasts in an environment with high glucose concentration (40 mM) showed significantly elevated Cx43 expression. The expression of Cx43 was also shown in fibroblasts taken from human tissues within the diabetic foot ulcer was significantly higher than in the intact skin, both in patients with and without DM [124].

Therefore, the question of the influence of DM on the development of resistance to paclitaxel is interesting and, on the other hand, requires further work in the field of basic research. It is also an interesting direction for research that assesses the clinical importance of these mechanisms in the ISR process in patients living with DM.

### 6.2. mTOR Inhibitors

The mammalian target of rapamycin (mTOR) forms two complexes: mTORC1 (mainly regulating cell growth and metabolism) and mTORC2 (mainly responsible for the control of cell proliferation and survival). mTOR is involved in signalling pathways such as the PI3K/Akt pathway, tuberous sclerosis complex subunit 1/tuberous sclerosis complex subunit 2/Rheb (the TSC1/TSC2/Rheb pathway), liver kinase B1/adenosine 5′-monophosphate-activated protein kinase (the LKB1/AMPK pathway), and VAM6/Rag GTPases [125]. Interestingly, the role of mTORC1 in the development of insulin resistance, which is the key mechanism in the pathogenesis of T2DM, has been emphasized. The chronic activation of mTORC1 is associated with an excessive accumulation of adipose tissue, including visceral fat. Zoncu et al. pointed out that the chronic activation of mTORC1 promotes the development of insulin resistance and that insulin activates mTORC1. The constitutive activity of mTOR is probably due to the increased concentration of amino acids in the blood present in obesity [126]. Such mechanism defects of mTOR-regulated proteins (S6K1, 4E-BP1, PP2A-related phosphatases, and p27), as well as the status of ATM, p53, PTEN/Akt and 14-3-3 protein, may be responsible for resistance to sirolimus.

However, the clinical significance of resistance to mTOR inhibitors in ISR is not well understood [25]. It may be an interesting direction for future research.

## 7. Genetic Factors

Table 5 contains examples of genes for which there are reports in the literature on the relationship between the presence of selected polymorphisms and the risk of restenosis. Data mainly come from studies of restenosis in coronary arteries. These genes encode proteins involved in the renin-angiotensin-aldosterone system, platelet aggregation, inflammatory response, intracellular matrix remodelling, smooth muscle cells proliferation, lipid metabolism, NO metabolism, and oxidative stress [127].

Although research on the influence of gene polymorphisms on the risk of restenosis is very interesting, the significance of these phenomena has not been sufficiently established so far to be able to determine the standards for therapeutic management based on genetic tests. Sometimes, studies conducted by different teams of scientists give different results. For example, the results obtained by Monraats et al., Zee et al., and Shimada et al. on the influence of the CD14 encoded gene polymorphism on the restenosis process are not fully compliant [128,129,130].

Kaizer et al. showed that the expression of some genes may differ in patients with DM compared to people in the general population [131]. Therefore, it cannot be ruled out that the influence of the polymorphisms of the genes involved in ISR on its risk is different in patients with DM than in patients without DM. However, there are only a few studies available that only include people diagnosed with DM. Table 5 only lists genes for which the relationship of selected polymorphisms with the development of ISR has been investigated, along with the most interesting references from the literature. Only the results of studies focusing on ISR in patients with DM have been discussed in more detail.

**Table 5:** Selected genes whose polymorphism has been investigated to be involved in the modification of the risk of ISR.

Gene	Encoded Protein	Reference
*ACE*	angiotensin-converting enzyme	[132,133,134,135,136,137,138,139,140,141,142]
*AGT*	angiotensinogen	[132,134]
*ADRB2*	adrenergic β2-receptor	[128]
*GP Ia*, *GP IIb*, *GP IIIa*	platelets’ GP IIb/IIIa receptor and GP Ia/IIb receptor	[143,144,145,146,147]
*HP*	haptoglobin	[148]
*IL-1RN*	interleukin-1 receptor antagonist	[149,150,151]
*IL-1B*	interleukin-1 beta	[150]
*IL-10*	interleukin-10	[152,153]
*VEGF*	vascular endothelial growth factor	[154]
*CCL11*	eotaxin CCL11	[128]
*CSF2*	colony stimulating factor 2	[128]
*CD14*	cluster of differentiation 14 (CD14)	[128,129,130]
*eNOS*	endothelial nitric oxide synthase	[155,156,157]

Guneri et al. published the results of a study in which 130 individuals with CHD and T2DM participated. It has been documented that the use of angiotensin-converting enzyme inhibitors (ACEI) in patients with D allele is significantly associated with the risk of restenosis (ACEI ratio, 43.5% in the restenosis group and 56.5% in non-restenosis group, *p* < 0.05) [158]. Despite these results, in the meta-analysis prepared by Kitsios et al., it was shown that there is no evidence suggesting that genetic testing of the ACE I/D polymorphism prior to clinical decision making would be justified [159].

Gazzaruso et al. demonstrated in a prospective study, in which only patients with T2DM had participated, that, between patients with and without ISR, significant difference in apolipoprotein(a) [apo(a)] exists in univariate analysis. Logistic regression analysis showed it not to be an independent risk factor. Only multivessel disease has been found to be a predictor of ISR after coronary stent implantation [160]. The same research team obtained similar results in another study that was not limited to people with DM [161]. The apo(a) polymorphism is determined by the number of repeats of kringle IV type 2 (KIV-2) within the *Lpa* gene and is related to the size of the molecule [162].

## 8. Technical and Mechanical Factors

Stent under-expansion, stent fracture, over-dilatation, non-uniform drug deposition, non-uniform stent strut distribution, and polymer damage are mechanical factors increasing the risk of ISR. Barotrauma outside the stented segment, residual uncovered atherosclerotic plaque, and stent gap are technical factors that increase the risk of ISR [25].

Due to mobility and associated mechanical stresses within the stent, mechanical factors seem to be particularly important in the area of the lower limbs. Mazzaccaro et al. presented a biomechanical model showing the phenomenon of stent fatigue failure within SFA and its translation into the phenomenon of stent fracture [163]. Interestingly, it has been recently shown that an oversized stent is associated with a significantly increased risk of ISR in SFA after self-expanding nitinol stent implantation. In the study group, the stent-to-artery diameter ratio was significantly higher in the distal stent than in the proximal stent (1.55 ± 0.25 vs. 1.3 ± 0.2, *p* = 0.001). Similarly, the risk of ISR was significantly higher in the distal stent than in the proximal stent (52.6% vs. 37.3%, *p* = 0.029) [164].

Interestingly, DES overlap leads to an increased risk of adverse clinical events after PCI. Although at 10 years, all-cause mortality did not differ between the stent overlap and no stent overlap groups, myocardial infarction (8.4% vs. 5.2%, *p* < 0.001) and target lesion revascularisation (23.7% vs. 16.3%, *p* < 0.001) occurred significantly more frequently in the group with stent overlap [165].

The main topic of our work is the process of restenosis in patients with DM, and there are no indications that the mechanical and technical factors related to stent implantation play a greater role in this population than in the general population, so we only signal the role of these mechanisms in the restenosis process, with no further development this topic.

## 9. Clinical Significance of Restenosis

In 2020, Moussa et al. presented the results of the analysis of data from 5,100,394 PCI procedures from approximately 1400 catheterisation laboratories in the United States of America, which show how clinically important restenosis is. Out of this number of PCI procedures, 10.6% were performed due to ISR. A clinical picture of unstable angina was significantly more frequent in patients with ISR than without ISR (51.8% vs. 38.6%, *p* < 0.001). Furthermore, 18.7% of individuals with ISR developed non-ST-elevation myocardial infarction (NSTEMI), and 8.5% of subjects with ISR suffered from STEMI [166].

Van Belle et al. presented the results of a six-month follow-up study in which it was found that, although the rate of restenosis after balloon angioplasty without stent implantation in the coronary arteries is significantly higher in patients with DM (63% vs. 36%, *p* = 0.0002), there is no difference between the incidence of ISR between subgroups with and without DM [22]. Perhaps the observation period here was too short, and therefore, no effect of DM on the frequency of ISR was found. In a one-year follow-up study performed by Paramasivam et al., it was shown that, in subjects with DM, the incidence of stent-edge restenosis after PCI with DES implantation is higher than in individuals without DM (20.3% vs. 9.2%, *p* = 0.019) [167].

PCI for ISR lesions is associated with similar clinical outcomes compared to PCI for de novo lesions. A study by Takeuchi et al. was a retrospective analysis of 1538 patients who were treated with PCI between 2013 and 2020. In two-year follow-up, both the risk of major adverse cardiac and cerebrovascular events (HR 1.10, 95% CI 0.49–2.49; *p* = 0.81), as well as all-cause mortality (HR 0.58, 95% CI 0.26–1.31; *p* = 0.19), were not significantly different between groups, although patients in the group with ISR were significantly older, with a higher prevalence of hypertension, DM, dyslipidemia, and chronic kidney disease [168].

The scale of the problem of ISR in the endovascular treatment of PAD is well demonstrated by the results of a study conducted by Tan et al. The study retrospectively analysed 260 femoropopliteal lesions treated in 250 patients with the implantation of LifeStent self-expanding nitinol stents. The 3-year restenosis rate for the total population was estimated at 72.9%, and serious limb adverse events were reported in 36.9% [169]. In another study, the outcomes of the endovascular treatment of 481 femoropopliteal lesions with the implantation of InnovaTM self-expanding nitinol stents were analysed in 453 patients. In one-year follow-up, restenosis occurred in 36% and a major adverse limb event in 18%. The rate of people living with DM in the study population was 61% [170].

It has recently been shown that, in patients living with DM treated with the infrapopliteal endovascular revascularisation, poor periprocedural glycaemic control is associated with a higher risk of restenosis, whereas post-procedural dual-antiplatelet therapy was found to be an independent predictor of amputation-free survival [171].

In the multicenter RELIABLE study, 77 Japanese individuals with target-lesion stenosis, restenosis (≥50% of the diameter of the reference vessel), occlusion in the SFA or an infrapopliteal runoff vessel to the foot (in at least one patient) were enrolled. A previously implanted stent at the proposed treatment site was one of the exclusion criteria. PTA with nitinol self-expanding second-generation stent placement was performed. The primary patency rate was 71.0% at 12 months and 67.8% at 36 months. Primary patency has been defined as freedom from the restenosis of the target lesion (luminal narrowing of ≥50%), determined by angiography or ultrasonography, and/or the revascularisation of the target lesion [172].

Angioplasty with DCB reduces the frequency of restenosis. In the prospective randomised RANGER SFA (Comparison of the Ranger™Paclitaxel-Coated PTA Balloon Catheter and Uncoated PTA Balloons in Femoropopliteal Arteries) study, 105 patients participated with symptomatic lower limb ischemia and without history of stent implantation in femoropopliteal segment. Patients with DM accounted for 35% of the control group and 39% of the research group. At 12 months, the primary patency rate was significantly higher for the Ranger DCB group than in patients who underwent angioplasty with a control balloon (86.4% [95% CI 78.5–95.1%] vs. 56.5% [95% CI 41.1–77.6%], *p* < 0.001) [173].

It has been recently documented (PERMIT-ISR Trial) that mechanical atherectomy plus thrombectomy using the RotarexS device followed by PTA with DCB can be a safe and effective treatment method for femoropopliteal ISR. The freedom rate from target lesion revascularisation was 84.7% at one year. The Rutherford category and the value of ABI at 12 months were significantly improved when compared to the baseline (*p* < 0.01). Subjects with DM accounted for 45.8% of the study population [174].

## 10. Conclusions

As a result of our detailed review of the literature, we have described the most current information on the pathogenesis and clinical significance of the phenomenon of restenosis. In our opinion, the information collected in this study may be helpful in planning further research on restenosis, which may contribute to the development of more and more precise recommendations for clinical practice.

Restenosis, also taking into consideration ISR, is an important issue that is of great interest to clinicians and researchers, and thus, knowledge on this subject is dynamically developing. In recent years, some well-prepared reviews have been published that widely describe the mechanisms and clinical significance of restenosis. However, the literature review presented by us is distinguished by the fact that special attention has been paid to the issue of restenosis in patients with DM, and a great deal of emphasis has been placed on restenosis after interventional treatment of PAD, although many of the phenomena that make up this process are similar within the coronary arteries and the peripheral arteries. The most important findings of our review of the literature are summarised in the Table 6.

While searching the literature on restenosis for the purposes of this review, attention was drawn to a clear disproportion in the available literature on invasive treatment of coronary and peripheral arteries. PAD is a serious medical problem that may lead to amputation, poor quality of life, disability, and premature death. According to Pawlik et al., in patients with CTO, the rate of individuals with coexisting CHD and chronic obstructive pulmonary disease (COPD) is significantly higher in males, whereas the rate of individuals with DM and hypertension is significantly higher in females. Comorbidities make PAD treatment more difficult, and it is necessary to take into consideration the general clinical picture [175]. Patients with comorbidities such as CHD and COPD are more likely to experience more advanced and multi-level atherosclerotic lesions [176]. DM is the strongest risk factor for PAD development, so the diagnosis and treatment of PAD in patients living with DM is extremely important [9]. Patients with DM should be closely monitored for the development of CLI and, if suspected, under the care of a specialist in vascular diseases [177].

It may be concluded that every effort should be made to develop knowledge about the pathogenesis of restenosis, including ISR, for the endovascular treatment of both CHD and PAD, which may lead to the availability of more and more perfect therapeutic methods in clinical practice.

## Figures and Tables

**Table 1:** Selected differences and similarities between neoatherosclerosis and atherosclerosis in native arteries.

There are focal calcifications at sites where macrophage apoptosis has occurred, both in neoatherosclerosis and in atherosclerosis in native arteries [31,32].
Atherosclerosis of native arteries takes many years to develop, in contrast to neoatherosclerosis, which takes months or a few years to develop [33].
VSMCs proliferation without macrophages infiltration is typical for in-stent restenosis (within BMSs) [33].
Calcified nodules, which are a relatively rare cause of thrombosis in the atherosclerosis of the native arteries, have not yet been observed in neoatherosclerosis [33].
Unlike the atherosclerosis of the native arteries, macrophages within neoatherosclerosis in stents tend to accumulate as superficial aggregates or in the per strut regions [31].
Neoatherosclerosis is typically identified by macrophage foam cell infiltration, intraplaque haemorrhage, and a thin fibrous cap [33].

**Table 2:** Selected differences and similarities between neoatherosclerosis within BMSs and DESs.

The analysis of 299 autopsies showed the incidence of neoatherosclerosis to be significantly greater in DESs (31%) than in BMSs (16%) (*p* < 0.001) [34].
Neoatherosclerosis occurs earlier in DESs (420 days (361–683)) in comparison to BMSs (2160 days (1800–2880)) (*p* < 0.001) [34].
Early neointima in DES consists of peristrut fibrin with a small amount of VSMCs within a proteoglycan-rich extracellular matrix with poor strut endothelialisation [35].
Neointima in BMS is relatively thick. It is mainly composed of VSMCs in a proteoglycan/collagenous matrix with endothelial coverage within 3 to 4 months [30].
In the case of stents implanted less than two years ago, in neoatherosclerosis within DES, there is a greater incidence of foamy macrophage clusters, as well as fibroatheromas [34].
It has been shown that neoatherosclerosis in BMSs occurs more frequently in the proximal than in the middle or distal segment. For DESs, no such difference was found [34].

**Table 3:** The most important atherogenic mechanisms in patients with DM.

Inflammation	Increased activity of C-reactive protein (CRP) and proinflammatory cytokines
Endothelial dysfunction	Hyperglycaemia, insulin resistance, and free fatty acid production decrease nitric oxide (NO) bioavailability [36,37].
	Hyperglycaemia worsens endothelial nitric oxide synthase (eNOS) function [36,37].
Platelets’ dysfunction	Upregulation of P-selectin, GP Ib receptor and GP IIb/IIIa receptor [12,38]
	Activation of protein kinase C (PKC) and decrease in NO production [12]
	Enhanced platelet adhesion and aggregation [12]
Coagulation	Upregulation of VIIa factor and tissue factor, downregulation of antithrombin III, protein S, and protein C [39,40,41]
	Hypercoagulability—according to the mechanisms elucidated above
Rheology	Elevated blood viscosity [12]
	Elevated fibrinogen production [12]
VSCMs	Promotion of the atherogenic phenotype of VSMCs through the increased production of reactive oxygen species, upregulation of PKC, advanced glycation end product receptors and nuclear factor kappa-light-chain-enhancer of activated B cells (NF-κB) [12]
	Impaired synthesis of collagen (plaque instability) [12]
	Increased activity of MMPs [42]
	Increased activity of angiotensin II and endothelin-1 (vasoconstriction) [12,43]

**Table 4:** Mechanisms of direct influence of CRP and proinflammatory cytokines on atherogenesis.

CRP promotes the production of procoagulant tissue factor, leukocyte adhesion molecules, and chemotactic substances [12].
CRP inhibits eNOS, which is associated with derangement in vascular tone [10].
CRP stimulates the production of plasminogen activator inhibitor-1 (PAI-1), which is associated with impaired fibrinolysis [12].
TNF-α and IL-6 via binding to receptors on the endothelial cell surface activate NF-κB and promote the transcription of genes encoding cell adhesion molecules, leading to the increased adhesion of white blood cells and platelets to the endothelium [12].

**Table 6:** Summary of the most important findings of our review of the literature.

Although the introduction of DESs into the clinical practice was a significant advance, restenosis remains an important factor limiting the effectiveness of the percutaneous revascularisation.
The inflammatory process in response to vascular injury plays an important role in the pathogenesis of restenosis. The role of the complement system seems to be an interesting direction for future research in patients with DM. The ratio of the number of neutrophils to lymphocytes in the peripheral blood has prognostic value for the risk of restenosis in patients treated endovascularly.
Inflammation, endothelial dysfunction, platelets’ dysfunction, coagulation, rheology, and smooth muscle cell proliferation in patients living with diabetes have a slightly different course, thus playing a special role in the pathogenesis of atherosclerosis.
The expression of Cx43, the TWEAK/Fn14 pathway, the AMPK/Nox4 pathway, the PI3K/Akt pathway, the activation of PKG by the translocator protein, and the increased expression of BMP-2 are examples of cellular phenomena involved in the smooth muscle cell proliferation process in patients with diabetes, which contributes to neointimal hyperplasia.
The mechanisms by which patients with DM may develop resistance to drugs used to coat drug-eluting stents (mTOR kinase inhibitors and paclitaxel) have been described. The research conducted so far mainly concerns the use of these drugs in the chemotherapy of malignant neoplasms. The influence of individual cytostatic resistance on the predisposition to restenosis in coated stents is an interesting direction for future research.
The process of allergic inflammation may also play a role in the development of restenosis.
The role of genetic polymorphisms is an interesting and promising direction in the research on the pathogenesis of restenosis. However, for now, the knowledge on this subject is not developed enough to allow genetic testing to influence the routine medical practice in the context of restenosis prevention.

## Data Availability

We used PubMed and Web of Science to screen articles for this narrative review. We did not report any data.

## Abbreviations

ABI	ankle–brachial index
ACEI	angiotensin-converting enzyme inhibitors
AMPK	AMP-activated protein kinases
apo(a)	apolipoprotein(a)
BCRP	breast cancer resistance protein
BMP	bone morphogenetic protein
BMS	bare-metal stent
cAMP	cyclic adenosine monophosphate
C5aR1	the first receptor for complement anaphylatoxin C5a
C5aR2	the second receptor for complement anaphylatoxin C5a
CAR	C-reactive protein-to-albumin ratio
CHD	coronary heart disease
CI	confidence interval
CLI	critical limb ischemia
COPD	chronic obstructive pulmonary disease
CREB	cAMP response element-binding protein
CRP	C-reactive protein
CRT3	CREB-regulated transcriptional coactivator 3
CTO	chronic total occlusion
Cx43	connexin 43
DCB	drug-coated balloon
DES	drug-eluting stent
DM	diabetes mellitus
EC	endothelial cell
ECP	eosinophil cationic protein
eNOS	endothelial nitric oxide synthase
GJ	gap junction
HIF	hypoxia-inducible factor
HR	hazard ratio
hs-CRP	high-sensitivity C-reactive protein
IgE	immunoglobulin E
IL	interleukin
IMT	intima-media thickness
IP_3_	inositol 1,4,5-trisphosphate
ISR	in-stent restenosis
KIV-2	kringle IV type 2
LKB1	liver kinase 1
LMR	lymphocyte-to-monocyte ratio
MAPK	mitogen-activated protein kinase
MMP	matrix metalloproteinase
mTOR	mammalian target of rapamycin
mTORC1	mammalian target of rapamycin complex 1
mTORC2	mammalian target of rapamycin complex 2
NF-κB	nuclear factor kappa-light-chain-enhancer of activated B cells
NLR	neutrophil-to-lymphocyte ratio
NLRP3	the NOD-, LRR-, and pyrin domain-containing protein 3
NO	nitric oxide
NSTEMI	non-ST-elevation myocardial infarction
OR	odds ratio
PAD	peripheral arterial disease
PAI-1	plasminogen activator inhibitor-1
PCI	percutaneous coronary intervention
PDGF	platelet-derived growth factor
PGK1	phosphoglycerate kinase 1
PI3K	phosphatidylinositol 3-kinase
PKA	protein kinase A
PKB	protein kinase B
PKC	protein kinase C
PKG	protein kinase G
PLR	platelet-to-lymphocyte ratio
PTA	percutaneous transluminal angioplasty
SFA	superficial femoral artery
SHP-1	Src homology-2-containing protein tyrosine phosphatase
SIK	salt-inducible kinase
STAT3	signal transducer and activator of transcription 3
STEMI	ST-elevation myocardial infarction
T1DM	type 1 diabetes mellitus
T2DM	type 2 diabetes mellitus
TGF-β	transforming growth factor beta
TLR4	toll-like receptor 4
TNF-α	tumour necrosis factor alpha
TSC	tuberous sclerosis complex
TWEAK	tumour necrosis factor-like weak inducer of apoptosis
VCAM-1	vascular cell adhesion molecule-1
VSMC	vascular smooth muscle cell
WT	wild-type
